# School and Work Absences After Critical Care Hospitalization for Pediatric Acute Respiratory Failure: A Secondary Analysis of a Cluster Randomized Trial

**DOI:** 10.1001/jamanetworkopen.2021.40732

**Published:** 2021-12-23

**Authors:** Erin F. Carlton, John P. Donnelly, Hallie C. Prescott, Lisa A. Asaro, Ryan P. Barbaro, R. Scott Watson, Martha A. Q. Curley

**Affiliations:** 1 Division of Critical Care Medicine, Department of Pediatrics, University of Michigan, Ann Arbor; 2 Susan B. Meister Child Health Evaluation and Research Center, Department of Pediatrics, University of Michigan, Ann Arbor; 3 Department of Learning Health Sciences, University of Michigan Medical School, Ann Arbor; 4 Veterans Affairs Center for Clinical Management Research, Health Service Research and Development Center of Innovation, Ann Arbor, Michigan; 5 Division of Pulmonary and Critical Care, Department of Internal Medicine, University of Michigan, Ann Arbor; 6 Department of Cardiology, Boston Children’s Hospital, Boston, Massachusetts; 7 Division of Pediatric Critical Care Medicine, Department of Pediatrics, University of Washington, Seattle; 8 Center for Child Health, Behavior, and Development, Seattle Children’s Research Institute, Seattle, Washington; 9 Family and Community Health, School of Nursing, University of Pennsylvania, Philadelphia; 10 Anesthesia and Critical Care Medicine, Perelman School of Medicine, University of Pennsylvania, Philadelphia; 11 Research Institute, Children’s Hospital of Philadelphia, Philadelphia, Pennsylvania

## Abstract

**Importance:**

Patients who survive pediatric critical illness and their caregivers commonly experience physical, emotional, and cognitive sequelae. However, the rate and duration of school absence among patients and work absence among their caregivers are unknown.

**Objective:**

To determine the rates and duration of school absence among children who survived hospitalization with acute respiratory failure and work absence among their caregivers.

**Design, Setting, and Participants:**

The Randomized Evaluation of Sedation Titration for Respiratory Failure (RESTORE) cluster randomized trial included 2449 children from 31 sites to protocolized sedation (intervention) vs usual care (control) from June 6, 2009, to December 2, 2013. In total, 1360 children survived hospitalization and were selected for follow-up at 6 months after pediatric intensive care unit (PICU) discharge, which was completed from January 12, 2010, to April 13, 2015. This secondary analysis was conducted from July 1, 2020, to September 30, 2021.

**Exposures:**

PICU hospitalization for acute respiratory failure, including invasive mechanical ventilation.

**Main Outcomes and Measures:**

Postdischarge assessments with caregivers of eligible participants at 6 months after PICU discharge, including questions about school and work absence. Risk factors associated with longer absence from school and work were identified.

**Results:**

Postdischarge assessments were completed for 960 children who survived treatment for acute respiratory failure, of whom 443 (46.1%) were girls and 517 (53.9%) were boys; 509 of 957 (53.2%) were non-Hispanic White. Median age was 1.8 years (IQR, 0.4-7.9 years). In total, 399 children (41.6%) were enrolled in school, of whom 279 (69.9%) missed school after discharge. Median duration of postdischarge absence was 9.1 days (IQR, 0-27.9 days) among all children enrolled in school and 16.9 days (IQR, 7.9-43.9 days) among the 279 children with postdischarge absence. Among 960 primary caregivers, 506 (52.7%) were employed outside the home, of whom 277 (54.7%) missed work. Median duration of postdischarge work absence was 2 days (IQR, 0-10 days) among all employed primary caregivers, and 8 days (IQR, 4-20 days) among the 277 caregivers who missed work after discharge. The odds of postdischarge school absence and greater duration of absence increased for children 5 years or older (compared with 0-4 years, odds ratios [ORs] for 5-8 years, 3.20 [95% CI, 1.69-6.05] and 2.09 [95% CI, 1.30-3.37], respectively; ORs for 9-12 years, 2.49 [95% CI, 1.17-5.27] and 2.32 [95% CI, 1.30-4.14], respectively; and ORs for 13-18 years, 2.37 [95% CI, 1.20-4.66] and 1.89 [95% CI, 1.11-3.24], respectively) and those with a preexisting comorbidity (ORs, 1.90 [95% CI, 1.10-3.29] and 1.76 [95% CI, 1.14-2.69], respectively).

**Conclusions and Relevance:**

In this secondary analysis of a cluster randomized trial, 2 in 3 children hospitalized for acute respiratory failure missed school after discharge, for a median duration of nearly 2 weeks. In addition, more than half of primary caregivers missed work after discharge. The magnitude of school absenteeism suggests that children may be at increased risk for lower educational achievement, economic hardship, and poor health outcomes in adulthood.

## Key Points

**Question:**

How often and for how long do children and their caregivers miss school or work after critical care hospitalization for acute respiratory failure?

**Findings:**

In this secondary analysis of the Randomized Evaluation of Sedation Titration for Respiratory Failure clinical trial, more than two-thirds of children missed school after discharge for a median of 9.1 days. Among primary caregivers, more than half missed work after their child’s hospitalization for a median of 2 days.

**Meaning:**

These findings suggest that children and their caregivers often miss school or work after critical care hospitalization for acute respiratory failure; these children may be at increased risk of lower educational achievement, economic hardship, and poor health outcomes in adulthood.

## Teaser

This secondary analysis of a cluster randomized trial measures the rate and duration of school and work absence among children who survived hospitalization for acute respiratory failure and their caregivers, respectively.

## Introduction

Survivors of critical illness and their families commonly experience physical, emotional, and cognitive problems after hospitalization. These sequelae—termed postintensive care syndrome, or PICS^1^—have been increasingly recognized in survivors of pediatric critical illness.^2,3^ Because child and family health are closely entwined, deterioration in a child’s health has spillover effects onto the family, further impeding the child’s recovery and worsening their long-term health.^2,4^ Owing to the reciprocal relationship between child and family health, it is important to examine outcomes after critical illness not only among children, but also in their caregivers and siblings.^3,4^

Children and their families may also experience financial effects after critical illness, including inability of caregivers to return to work.^5,6^ Indeed, a recent meta-analysis found only 1 in 3 adults who survived critical illness was able to return to work within 3 months of discharge, and inability to return to work was associated with worse psychological outcomes.^7^ Similarly, children and their caregivers may have prolonged absence from school and work. However, the rate and duration of school and work absence after pediatric critical illness are unknown.

Thus, among participants in the Randomized Evaluation of Sedation Titration for Respiratory Failure (RESTORE) trial,^8,9^ we measured the rate and duration of school and work absence among children who survived pediatric intensive care unit (PICU) hospitalization for acute respiratory failure and their caregivers, respectively. In addition, we identified patient, family, and hospitalization characteristics associated with prolonged school or work absence.

## Methods

### Patients

RESTORE, a 31-center cluster randomized trial,^8^ enrolled 2459 patients aged 2 weeks to 17 years from June 6, 2009, to December 2, 2013; consent was withdrawn for 10, leaving 2449 patients. Inclusion criteria included invasive mechanical ventilation for acute respiratory failure. Each site obtained institutional review board approval and written informed consent from the legal guardians of patient participants. Parents and guardians provided consent during acute hospitalization for follow-up at 6 months after PICU discharge. Telephone follow-up assessments were conducted on a sample of participants stratified by age and site. In total, 1360 patients survived hospitalization and were selected for follow-up.^10,11^ Thirty patients died before follow-up, leaving 1330 patients eligible for follow-up at 6 months, completed from January 12, 2010, to April 13, 2015. This secondary analysis was conducted from July 1, 2020, to September 30, 2021. Our study follows the Strengthening the Reporting of Observational Studies in Epidemiology (STROBE) reporting guidelines.

### Primary Outcomes

During the 6-month postdischarge survey, caregivers were asked about school enrollment, school absence, employment, and work absences among children, siblings, and primary and secondary caregivers (Table 1 and eMethods 1 in Supplement 1). The primary outcomes of this study were school and work absences in the 6 months after discharge among patients who survived pediatric respiratory failure and their primary caregivers. Specifically, we measured (1) the rate and duration of absence from school, preschool, or day care (herein referred to as school) during the 6 months after discharge among patients who were enrolled in school before the hospitalization and (2) the rate and duration of work absence during the 6 months after discharge among primary caregivers who reported full-time or part-time employment outside of the home.

**Table 1.** Rate and Duration of School and Work Absences

Participant	Rate and duration^a^
**Child**	
Postdischarge school days	
Any missed days, No./total No. (%)	279/399 (69.9)
No. of missed days, median (IQR)	9.1 (0-27.9)
**Primary caregiver**	
Postdischarge work days	
Any missed days, No./total No. (%)	277/506 (54.7)
No. of missed days, median (IQR)	2 (0-10)
During hospitalization work days	
Any missed days, No./total No. (%)	426/506 (84.2)
No. of missed days, median (IQR)	10 (4-20)
**Secondary caregiver**	
Postdischarge work days	
Any missed days, No./total No. (%)	193/614 (31.4)
No. of missed days, median (IQR)	0 (0-2)
During hospitalization work days	
Any missed days, No./total No. (%)	484/614 (78.8)
No. of missed days, median (IQR)	5.5 (2-10)

^a^ Rate and duration of school or work missed were determined through the following survey questions: (1) Has your child missed any days of day care, preschool, or school since being admitted to the hospital roughly 6 months ago? (2) How many days of day care, preschool, or school has your child missed? (3) Since your child was discharged from the hospital, how many days have you had to stay home from work to be with him or her when you have been planning to work? and (4) Since your child was discharged from the hospital, how many days has another person who had been planning to work had to stay home from work to be with him or her?

We hypothesized that the rate and duration of school absence would be associated with the duration of hospitalization, so we focused our primary outcomes on absenteeism after discharge. Because the follow-up survey asked about total duration of school absence (inclusive of school missed during hospitalization), we calculated the rate and duration of postdischarge school absence among patients discharged during the school year (which we defined as September through June) by subtracting the estimated number of weekdays missed during hospitalization (5 weekdays per 7 days of hospitalization, described further in eMethods 2 in Supplement 1) from the total number of school days reported as missed.

### Secondary Outcomes

Secondary outcomes included the rate of chronic absenteeism after discharge (defined as missing >15 days of school)^12,13^ among patients who survived pediatric respiratory failure; the rate and duration of any school absence (either during hospitalization or after discharge); the rate and duration of work absence during hospitalization among primary caregivers; the rate and duration of work absence among secondary caregivers during hospitalization and after discharge; and the rate and duration of school absence among siblings during hospitalization and after discharge. As with the primary outcomes, the secondary outcomes were measured among at-risk individuals (ie, children enrolled in school and caregivers employed before hospitalization).

### Subgroup Analyses

We completed several subgroup analyses to better characterize the burden of school absence. First, to evaluate the association of acute respiratory failure with elementary, middle, and high school absence vs day care or preschool absence, we measured the rate and duration of postdischarge school absence among children 5 years or older and among children 4 years or younger. In addition, to estimate the association with part-time day care or preschool enrollment, we measured the rate and duration of postdischarge school absence assuming a planned attendance of 2 days per week for children 4 years or younger. To differentiate the association of acute hospitalization from baseline health, we measured the rate and duration of school absence among children by preexisting comorbidity status. Finally, to understand how a child’s age is associated with work absence, we evaluated rate and duration of postdischarge primary caregiver work absence by child age group (0-4, 5-9, 10-12, and 13-18 years).

### Statistical Analysis

To identify risk factors associated with school and work absenteeism, we completed several analyses. First, we evaluated whether the following characteristics differed across 4 groups defined by duration of absenteeism (no missed school/work and tertiles of missed school/work): patient characteristics (age, sex, race and ethnicity, preadmission functional status [Pediatric Overall Performance Category],^14^ and comorbidities); family characteristics (primary caregiver educational level and daily activity, secondary caregiver daily activity, median household income of zip code of residence); hospitalization characteristics (severity of illness [measured by Pediatric Risk of Mortality III],^15^ primary diagnosis, and PICU and hospital lengths of stay); and posthospitalization characteristics (hospital readmission, emergency department use after discharge). We evaluated trends in characteristics across these 4 groups using the χ^2^ test for trend or the nonparametric test for trend (Jonckheere-Terpstra test).^16^

Second, we fit a multivariable ordinal logistic (proportional odds) regression model for cumulative categories of duration of school absence (eg, longer or medium absence vs short or no absence), which included the following characteristics as risk factors associated with outcomes: age group (0-4, 5-9, 10-12, and 13-18 years), race and ethnicity, baseline functional impairment, prior comorbidity, admission diagnosis, and primary caregiver’s daily activity (full-time employment, part-time employment, homemaker, and other [ie, student, retired, or disabled]). In addition, we fit logistic regression models (yes or no absence) using the above risk factors.

We fit additional multivariable models including a dichotomous variable for the presence of a secondary caregiver and a count variable for the number of siblings to understand their impact on postdischarge school absence. Finally, to understand the association of the RESTORE intervention (protocolized sedation) with outcomes, we compared rates and duration of absence between the intervention and usual care arms by χ^2^ and Wilcoxon rank-sum tests. Because RESTORE was a cluster randomized trial, we also explored multilevel models (patients nested within sites) in preliminary analyses but found no appreciable site effect, so we present results without adjustment for site for simplicity.

The caregivers of 7 children did not recall whether they missed school, and a caregiver of 1 child did not respond to this question. For these children, we assumed no school was missed. In addition, caregivers of 10 children reported missed school but did not quantify the numbers of days missed. These children were excluded from analyses of postdischarge school absence. Statistical analyses were performed with Stata software, version 15 (StataCorp LLC). Two-sided *P* < .05 indicated statistical significance.

## Results

**Figure 1.**
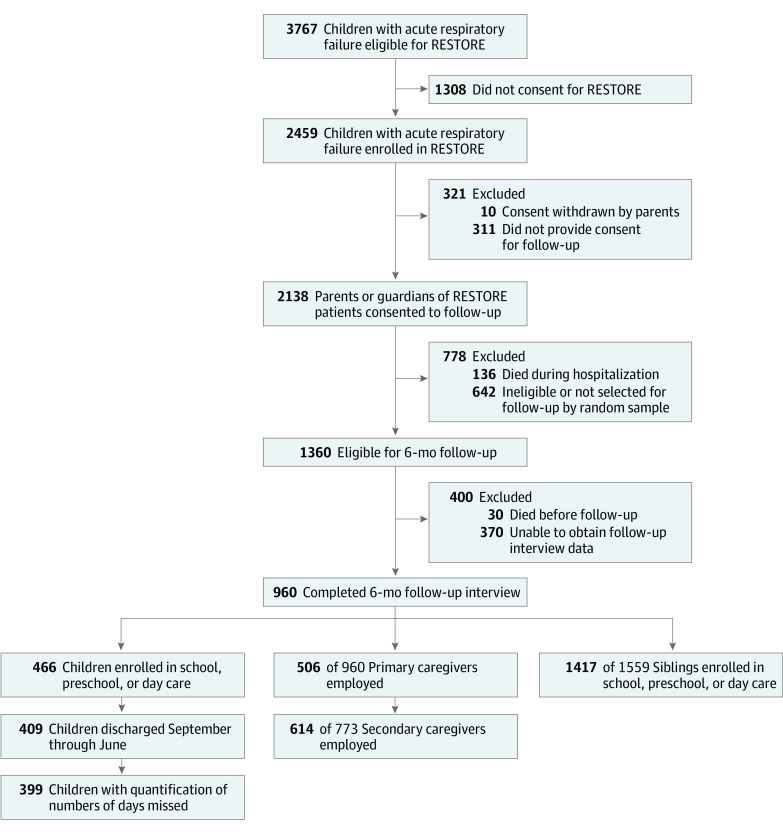
Flow Diagram of Study Participants RESTORE indicates Randomized Evaluation of Sedation Titration for Respiratory Failure.

Of 1330 patients enrolled in RESTORE and eligible and selected for 6-month postdischarge follow-up, 370 (27.8%) did not complete follow-up interviews (including 263 [19.8%] lost to follow-up) and 960 (72.2%) had a follow-up interview completed by a parent or guardian at a median of 6.9 months (IQR, 5.7-8.5 months) after discharge (Figure 1) and were included in this study. Demographic information was previously reported.^10^ Briefly, the cohort included 443 girls (46.1%) and 517 boys (53.9%). Race and ethnicity data were obtained from the medical record when available; if not available, research staff were instructed to ask families directly. In total, 33 of 957 patients (3.4%) with available data were Asian; 167 (17.5%), Black; 208 (21.7%), Hispanic/Latinx; 509 (53.2%), non-Hispanic White; and 40 (4.2%), other (American Indian or Alaska Native, Native Hawaiian or other Pacific Islander, and multiracial or >1 race and ethnicity).^12,17,18^ Median age was 1.8 years (IQR, 0.4-7.9 years).

### Child School Absence

**Figure 2.**
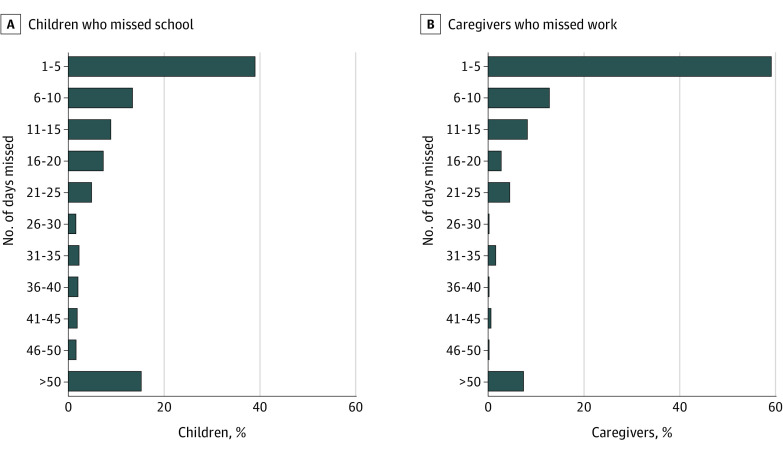
School and Work Days Absent in the 6 Months After Child’s Hospital Discharge Includes 399 children with quantification of days missed and 506 primary caregivers employed outside the home.

In total, 399 children (41.6%) were enrolled in school (defined as day care, preschool, or school) before hospitalization and discharged between September and June, of whom 279 (69.9%) missed school after discharge. Median duration of postdischarge school absence was 9.1 days (IQR, 0-27.9 days) among all children enrolled in school and 16.9 days (IQR, 7.9-43.9 days) among the 279 with any postdischarge absence (Table 1 and Figure 2). In total, 153 of 399 children (38.3%) met criteria for chronic absenteeism after discharge, missing more than 15 days of school. Among the 279 children with any postdischarge absence, 153 (54.8%) met criteria for chronic absenteeism.

Patient characteristics including older age, preexisting functional impairment, and preexisting comorbidity were more common among children with longer duration of postdischarge school absence (Table 2). Children with a longer median PICU length of stay had a significantly longer duration of postdischarge school absence (9.7 days [IQR, 6.0-16.5 days] for no absence vs 12.7 days [IQR, 6.8-19.3 days] for long absence; *P* = .04 for trend).

**Table 2.** Patient, Family, and Hospitalization Characteristics Across Duration of Postdischarge School Absence Category (N = 399)

Characteristic	School absence category^a^				*P* value^b^
	No absence	Short absence (n = 94)	Medium absence (n = 92)	Long absence (n = 93)	
Duration of school absence, median (IQR) [range], d	0 (0-0) [0-0]	5.7 (2.9-7.9) [0.3-10.0]	17.0 (13.6-22.1) [10.7-30.7]	64.3 (43.8-96.3) [31.7-219.8]	<.001
Patient characteristics					
Age at PICU admission, median (IQR), y	3.4 (0.6-10.1)	6.7 (4.4-10.9)	6.5 (3.4-12.3)	9.0 (5.8-12.5)	<.001
Sex					
Female	52 (43.3)	37 (39.4)	43 (46.7)	42 (45.2)	.59
Male	68 (56.7)	57 (60.6)	49 (53.3)	51 (54.8)	
Race and ethnicity					
Asian	7 (5.8)	4 (4.3)	1 (1.1)	0	.93
Black	22 (18.3)	18 (19.1)	21 (22.8)	17 (18.5)	
Hispanic/Latinx	16 (13.3)	12 (12.8)	11 (12.0)	13 (14.1)	
Non-Hispanic White	67 (55.8)	55 (58.5)	59 (64.1)	61 (66.3)	
Other^c^	8 (6.7)	5 (5.3)	0	1 (1.1)	
Missing, No.	0	0	0	1	
Functional impairment (POPC >1) at baseline^d^	28 (23.3)	21 (22.3)	33 (35.9)	42 (45.2)	<.001
Preexisting comorbidity					
Asthma	13 (10.8)	34 (36.2)	21 (22.8)	11 (11.8)	.89
Cancer	6 (5.0)	2 (2.1)	1 (1.1)	19 (20.4)	<.001
Chromosomal abnormality	4 (3.3)	3 (3.2)	6 (6.5)	10 (10.7)	.02
Prematurity	12 (10.0)	8 (8.5)	9 (9.8)	10 (10.7)	.82
Neurological/neuromuscular disorder	10 (8.3)	7 (7.4)	6 (6.5)	18 (19.3)	.02
Seizure disorder	7 (5.8)	11 (11.7)	14 (15.2)	17 (18.3)	.004
No known comorbidity	73 (60.8)	51 (54.3)	43 (46.7)	56 (60.2)	.009
Family characteristics					
Primary caregiver educational level					
Some high school	5 (5.5)	8 (11.3)	9 (13.6)	3 (4.8)	.77
High school graduate	18 (19.8)	17 (23.9)	18 (27.3)	14 (22.6)	
Some college or technical school	35 (38.5)	15 (21.1)	12 (18.2)	22 (35.5)	
College graduate/postgraduate	33 (36.3)	31 (43.7)	27 (40.9)	23 (37.1)	
Missing, No.	29	23	26	31	
Primary caregiver main daily activities					
Working					.47
Full-time	48 (40.3)	52 (55.9)	48 (53.3)	43 (46.7)	
Part-time	16 (13.4)	17 (18.3)	18 (20.0)	14 (15.2)	
Keeping house/raising children	42 (35.3)	17 (18.3)	17 (18.9)	27 (29.3)	
Other (ie, student, retired, disabled)	13 (10.9)	7 (7.5)	7 (7.8)	8 (8.7)	
Missing, No.	1	1	2	1	
Secondary caregiver main daily activities					
Working					.13
Full-time	74 (77.9)	52 (72.2)	58 (89.2)	61 (82.4)	
Part-time	7 (7.4)	8 (11.1)	2 (3.1)	4 (5.4)	
Keeping house/raising children	4 (4.2)	5 (6.9)	5 (7.7)	3 (4.1)	
Other (ie, student, retired, disabled)	10 (10.5)	7 (9.7)	0	6 (8.1)	
Missing, No.	25	22	27	19	
Household income of zip code of residence, median (IQR), $	55 521 (43 215-73 490)	62 691 (45 813-82 069)	56 315 (45 603-75 406)	63 571 (47 485-83 564)	.14
Income category of zip code of residence					
<$40 000	24 (20.0)	16 (17.0)	12 (13.0)	11 (11.8)	.13
$40 000-$79 999	70 (58.3)	51 (54.3)	59 (64.1)	56 (60.2)	
≥$80 000	26 (21.7)	27 (28.7)	21 (22.8)	26 (28.0)	
Duration of missed work of primary caregiver, median (IQR), d					
During hospitalization	8 (2-14)	8 (5-15)	15 (8-20)	20 (10-30)	<.001
After hospitalization	0 (0-5)	2 (0-5)	5 (0-10)	10 (0-39)	<.001
Hospitalization characteristics					
PRISM III-12 score, median (IQR)^e^	7.5 (5-11)	8 (4-13)	7 (3-14)	10 (5-16)	.10
Primary diagnosis category					
Bronchiolitis or asthma	38 (31.7)	32 (34.0)	22 (23.9)	6 (6.4)	<.001
Pneumonia or aspiration pneumonia	60 (50.0)	46 (48.9)	47 (51.1)	52 (55.9)	
Acute respiratory failure related to sepsis	13 (10.8)	11 (11.7)	12 (13.0)	22 (23.7)	
Other^f^	9 (7.5)	5 (5.3)	11 (11.9)	13 (14.0)	
Randomized to RESTORE sedation protocol	58 (48.3)	49 (52.1)	41 (44.6)	46 (49.5)	.87
PICU length of stay, median (IQR), d	9.7 (6.0-16.5)	7.3 (4.6-10.9)	10.4 (7.0-14.4)	12.7 (6.8-19.3)	.04
Functional impairment (POPC >1) at discharge^d^	39 (32.5)	26 (27.7)	48 (52.2)	54 (58.1)	<.001
Posthospitalization outcomes in 6 mo after discharge					
Emergency department use	44 (36.7)	24 (25.5)	30 (32.6)	42 (45.2)	.20
Hospital readmission	31 (25.8)	13 (13.8)	27 (29.3)	41 (44.1)	.002

Abbreviations: PICU, pediatric intensive care unit; POPC, Pediatric Overall Performance Category; PRISM III-12, Pediatric Risk of Mortality III score from first 12 hours in the PICU; RESTORE, Randomized Evaluation of Sedation Titration for Respiratory Failure.

^a^ Unless otherwise indicated, data are expressed as number (%) of patients. Percentages have been rounded and may not total 100.

^b^ Calculated for comparisons across categories using the χ^2^ test for trend or the nonparametric test for trend (Jonckheere-Terpstra test) for categorical and continuous variables, respectively.

^c^ Other includes American Indian or Alaska Native, Native Hawaiian or other Pacific Islander, and multiracial or more than 1 race and ethnicity.

^d^ Disability was determined using the POPC. A score of 1 indicates a good functional status, whereas a score of 2 or greater represents at least mild disability.

^e^ Uses physiologic and laboratory variables from the first 12 hours of PICU admission to calculate the overall risk of mortality. Scores range from 0 to 74, with higher scores representing a higher risk of mortality.

^f^ Includes pulmonary edema or hemorrhage, pulmonary hypertension, laryngotracheobronchitis, thoracic trauma, pneumothorax, acute chest syndrome, pertussis, exacerbation of lung disease (cystic fibrosis or bronchopulmonary dysplasia), acute respiratory failure related to blood transfusions, acute respiratory failure after bone marrow transplantation, and other.

In multivariable models, the odds of postdischarge school absence and greater duration of absence increased for children 5 years or older (compared to 0-4 years, odds ratios [ORs] for 5-8 years, 3.20 [95% CI, 1.69-6.05] and 2.09 [95% CI, 1.30-3.37], respectively; ORs for 9-12 years, 2.49 [95% CI, 1.17-5.27] and 2.32 [95% CI, 1.30-4.14], respectively; and ORs for 13-18 years, 2.37 [95% CI, 1.20-4.66] and 1.89 [95% CI, 1.11-3.24], respectively) and those with a preexisting comorbidity (ORs, 1.90 [95% CI, 1.10-3.29] and 1.76 [95% CI, 1.14-2.69], respectively) (Table 3). In the ordinal logistic regression model, baseline functional impairment was also associated with increased odds of longer durations of school absence (OR, 1.65; 95% CI, 1.05-2.60) (Table 3). Respiratory failure due to bronchiolitis or asthma was associated with lower odds of longer durations of school absence compared to respiratory failure due to pneumonia (OR, 0.54; 95% CI, 0.34-0.87) (Table 3). The presence of a secondary caregiver and number of siblings were not associated with rate of postdischarge school absence (eTable 1 in Supplement 1). The rates of postdischarge school absence (136 of 194 [70.1%] vs 143 of 205 [69.7%]; *P* = .94) and duration of postdischarge school absence (median, 8.9 days [IQR, 0-26.4 days] vs 9.3 days [IQR, 0-27.9 days]; *P* = .87) were similar among children randomized to protocolized sedation vs usual care (eTable 2 in Supplement 1).

**Table 3.** Multivariable Models of Risk Factors Associated With Rate and Duration of School Absence After Hospital Discharge

Variable	OR (95% CI)^a^	*P* value
Risk factors associated with rate of school absence (n = 392)^b^		
Age group, y		
0-4	1 [Reference]	.001
5-8	3.20 (1.69-6.05)	
9-12	2.49 (1.17-5.27)	
13-18	2.37 (1.20-4.66)	
Minority race or ethnicity	0.92 (0.56-1.51)	.74
Functional impairment (POPC >1) at baseline^c^	1.53 (0.83-2.80)	.17
Preexisting comorbidity	1.90 (1.10-3.29)	.02
Primary diagnosis category		
Pneumonia	1 [Reference]	.34
Bronchiolitis or asthma	0.80 (0.45-1.42)	
Acute respiratory failure related to sepsis	1.28 (0.61-2.71)	
Other	1.91 (0.77-4.74)	
Primary caregiver main daily activities		
Working full-time	1 [Reference]	.001
Keeping house/raising children	0.30 (0.17-0.55)	
Working part-time	0.79 (0.39-1.58)	
Other (student, retired, disabled)	0.47 (0.20-1.09)	
**Risk factors associated with category of duration of school absence (n = 392)^d^**		
Age group, y		
0-4	1 [Reference]	.005^d^
5-8	2.09 (1.30-3.37)	
9-12	2.32 (1.30-4.14)	
13-18	1.89 (1.11-3.24)	
Minority race or ethnicity	0.80 (0.54-1.18)	.26
Functional impairment (POPC >1) at baseline^c^	1.65 (1.05-2.60)	.03
Preexisting comorbidity	1.76 (1.14-2.69)	.01
Primary diagnosis category		
Pneumonia	1 [Reference]	<.001
Bronchiolitis or asthma	0.54 (0.34-0.87)	
Acute respiratory failure related to sepsis	1.58 (0.89-2.78)	
Other	2.29 (1.14-4.63)	
Primary caregiver main daily activities		
Working full-time	1 [Reference]	.12
Keeping house/raising children	0.57 (0.35-0.91)	
Working part-time	0.89 (0.53-1.47)	
Other (student, retired, disabled)	0.68 (0.33-1.37)	

Abbreviations: OR, odds ratio; POPC, Pediatric Overall Performance Category.

^a^ The largest group within each variable was chosen as the reference category.

^b^ An OR of greater than 1.0 indicates higher odds of missed school during hospital admission to 6 months after discharge. Odds ratios were calculated using logistic regression.

^c^ Disability was determined using the POPC. A score of 1 indicates a good functional status, while a score of 2 or greater represents at least mild disability.

^d^ An OR of greater than 1.0 indicates higher odds of missed school during hospital admission to 6 months after discharge. The OR describes the change in odds of increasing categories of duration of school absence, calculated using ordinal logistic (proportional odds) regression.

Of the 466 children enrolled in school (regardless of month of discharge), 384 (82.4%) missed school during hospitalization and/or after discharge, for a median of 20 days (IQR, 4-45 days). Among children who missed school, the median time missed during hospitalization and/or after discharge was 25 days (IQR, 12-60 days). Older age (median, 9.1 years [IQR, 5.9-12.5 years]), functional impairment (55 of 118 children [46.6%]), and preexisting comorbidity (eg, neurological/neuromuscular disorder, 22 of 118 children [18.6%]) were more common among children with longer duration of during-hospitalization and/or postdischarge school absence (eTable 3 in Supplement 1).

### Primary Caregiver Work Absence

Of 960 primary caregivers, 506 (52.7%) were employed before the hospitalization, of whom 277 (54.7%) missed work after discharge. Median duration of postdischarge work absence was 2 days (IQR, 0-10 days) among all employed primary caregivers and 8 days (IQR, 4-20 days) among the 277 who missed work after discharge.

A child’s preexisting comorbidity (eg, cancer, 13 of 84 [15.5%]), functional disability at hospital admission (32 of 84 [38.1%]) and discharge (48 of 84 [57.1%]), and longer PICU length of stay (median, 12.3 days [IQR, 7.3-19.1 days]) were more common among primary caregivers with longer duration of postdischarge work absence (eTable 4 in Supplement 1). Duration of postdischarge work absence among primary caregivers was associated with duration of postdischarge school absence. Among children with no school absence after discharge, their primary caregivers missed work for a median of 0 days (IQR, 0-5 days) after discharge, compared with a median of 10 days (IQR, 0-39 days) for primary caregivers of children who missed the most days of school (*P* < .001 for trend) (Table 2).

Rate of primary caregiver postdischarge work absence (142 of 269 [52.8%] vs 135 of 237 [57.0%]; *P* = .35) and duration of postdischarge work absence (median, 2 days [IQR, 0-10 days] vs 2 days [IQR, 0-10 days]; *P* = .61) were similar between caregivers of children in the sedation protocol vs usual care group (eTable 2 in Supplement 1). In total, 426 primary caregivers (84.2%) missed work during their child’s hospitalization. Median duration of during-hospital work absence was 10 days (IQR, 4-20 days) among all previously employed primary caregivers, and 10 days (IQR, 6-20 days) among the 426 with any during-hospital absence.

### Secondary Caregiver Work Absence

Among 773 secondary caregivers, 614 (79.4%) were employed before the hospitalization, of whom 193 (31.4%) missed work after discharge. Median duration of postdischarge work absence was 2 days (IQR, 0-2 days) among all employed secondary caregivers and 4 days (IQR, 2-8 days) among the 193 who missed work after discharge. Child functional impairment at admission (28 of 58 [48.3%]) and discharge (29 of 58 [50.0%]) were more common among secondary caregivers with longer duration of postdischarge work absence (eTable 5 in Supplement 1).

In total, 484 secondary caregivers (78.8%) missed work during hospitalization. Median duration of during-hospital work absence was 6 days (IQR, 2-10 days) among all employed secondary caregivers and 8 days (IQR, 5-14 days) among the 484 who missed work during hospitalization.

### Sibling School Absence

Of 960 children with acute respiratory failure, 790 (82.3%) had a total of 1559 siblings, of whom 1417 (90.9%) were enrolled in school. Only 66 (4.7%) of the school-enrolled siblings missed school after discharge. The median duration of school absence after discharge was 0 days (IQR, 0-0 days) and 2 days (IQR, 4-10 days) for those with postdischarge school absence. In total, 282 siblings (19.9%) missed school during hospitalization. The median duration of school absence during hospitalization was 0 days (IQR, 0-0 days) among all siblings enrolled in school and 3 days (IQR, 2-6 days) for children with during-hospital school absence.

### Subgroup Analyses

Of the 399 children enrolled in school, 247 (61.9%) were 5 years or older, of whom 196 (79.4%) missed school after discharge. Median duration of postdischarge school absence was 13.3 days (IQR, 1.6-42.1 days) among all children 5 years or older enrolled in school and 19.6 days (IQR, 8.6-55.0 days) among the 196 with any postdischarge absence (eTable 6 in Supplement 1). In total, 152 children enrolled in school were 4 years or younger, of whom 83 (54.6%) missed school after hospital discharge. Median duration of postdischarge school absence was 3.6 days (IQR, 0-14.6 days) among all children 4 years or younger enrolled in school and 12.9 days (IQR, 7.1-22.9 days) among the 152 with any postdischarge absence (eTable 6 in Supplement 1). In our analysis examining a 2-day school week for children 4 years and younger, 98 (64.5%) missed school. Median duration of postdischarge school absence was 9.3 days (IQR, 0-21.9 days) among all children 4 years or younger enrolled in school and 17.0 days (IQR, 9.3-27.6 days) among the 98 with any postdischarge absence.

Among 202 school-enrolled children without preexisting comorbidity, 129 (63.9%) missed school after discharge. Median duration of postdischarge school absence was 6.5 days (IQR, 0-20.7 days) overall and 16.4 days (IQR, 7.9-34.3 days) among the 129 with any postdischarge absence. Among 197 school-enrolled children with preexisting comorbidity, 150 (76.1%) missed school after discharge. Median duration of postdischarge school absence was 10.7 days (IQR 1.0-40.9 days) overall and 17.1 days (IQR, 7.9-57.9 days) among the 150 with any postdischarge absence. Rates of postdischarge work absenteeism among primary caregivers did not differ by child age category (eTable 7 in Supplement 1).

## Discussion

In this longitudinal follow-up of the RESTORE trial cohort, 69.9% of patients who survived pediatric acute respiratory failure missed school (broadly defined as school, preschool, or day care) after discharge. Furthermore, 54.7% of primary caregivers missed work after their child’s hospitalization. Importantly, the durations of school and work absenteeism were substantial—a median of nearly 2 five-day school weeks (9.1 days) of postdischarge school absence for children and a median of 1.5 five-day work weeks (8 days) of postdischarge work absence for primary caregivers who missed work. For children 5 years or older, the median duration was even longer (13.3 days). Among children who missed school, the duration of school absenteeism commonly exceeded the threshold for chronic absenteeism (>15 days missed), which is known to be associated with increased risk for a number of negative outcomes, including reduced academic achievement, economic hardship, and poor health outcomes in adulthood.^12^ These findings suggest that patients who survive pediatric respiratory failure are at increased risk for long-term sequelae not only due to their acute illness but also as a result of school missed during and after hospitalization.

Our study also examined school and work absence among siblings and secondary caregivers, respectively. Only 1 in 5 siblings missed school during the hospitalization, and the duration of absence was modest (median, 3 days). Furthermore, absence after hospitalization was rarer (occurring in only 4.7% of siblings). The rate and duration of absence among secondary caregivers, however, were nearly as high as those among primary caregivers.

As would be expected, worse child health status (eg, preexisting comorbidity, functional impairment) was associated with a greater burden of school and work absence. However, even among children without preexisting comorbidity, almost 63.9% missed school for a median duration of 6.5 days. Unlike prior studies,^19,20^ we did not find an association between neighborhood income and missed school or work among children or caregivers.

Consistent with prior studies, we found that rates of postdischarge absence differed by hospital diagnosis. Respiratory failure due to asthma or bronchiolitis was associated with a lower burden of school absence than respiratory failure due to pneumonia, sepsis, or other causes. Previous studies have shown that hospitalization for acute medical conditions,^21^ including infections,^22^ burns,^23^ transplant,^24^ and diabetes,^25^ are associated with subsequent absenteeism and lower academic achievement. Similarly, chronic health conditions and special health care needs (eg, prescription medications, specialized medical care, or educational services) are associated with school absence.^26,27,28^

Although it is not surprising that children would miss school during critical illness, the duration of school absence experienced by some children in our study is sufficient to result in long-term academic impairment. School absence in childhood has been associated with worse long-term socioeconomic and health outcomes, including school dropout, depression, substance abuse, and economic hardships in adulthood.^12,29,30^ The importance of school attendance begins before elementary school, because absence from preschool has been associated with lower levels of kindergarten readiness and increased risk of needing reading intervention by second grade.^31^ A recent analysis estimated that 5.5 million years of life were potentially lost owing to COVID-19–related school closures during 2020 alone, underscoring the importance of school attendance on health outcomes in adulthood.^32^ Importantly, the longer the duration of school absence, the greater the risk of impaired academic achievement. Chronic absenteeism (missing >15 days of school)^13^ is an important threshold associated with lower academic achievement and higher participation in risky health behaviors.^12^ More than half of the children in our study who missed school after discharge met criteria for chronic absenteeism.

Our study suggests that post-PICU school absenteeism is an important target for future interventions. Although school absenteeism is common after hospitalization and consistently associated with a multitude of negative outcomes, a recent study^33^ suggested that pediatricians ask about school absence in fewer than half of visits after hospitalization. Future work is needed to understand the barriers to school participation, to develop interventions to mitigate absenteeism, and to help children catch up on missed school.

Consistent with prior studies,^4,34,35,36^ and not unexpectedly given continued recovery after discharge, caregivers in our study commonly missed work after their child’s hospitalization. Notably, however, our study does not account for changes in informal caregiving practices at home, nor does it quantify the financial cost of missed work. In our cohort, work absence occurred during and after hospitalization and was strongly associated with the duration of child school absence, creating the potential for additive or multiplicative stress on families. Prior studies show 1 in 3 parents worry about job loss or reduced wages when taking time off to care for a sick child^37^ and that child health conditions are associated with lower parental employment.^38,39^ Even with employer-based sick leave, paid time off, and federal family leave programs, parents commonly report being unable to miss work for child illness.^40^ Indeed, only 56% of US employees are eligible for Family Medical Leave Act protections, which guarantee employment protection but do not guarantee wages.^41^ Parents with access to leave or paid benefits were more likely to miss work when needed by their child, suggesting awareness and access to family leave benefits may decrease this stressful conflict.^40^ Thus, given the magnitude of missed work found in our study and the hardships described by parents in prior studies, there is a great need for programs and policies to support families during and after pediatric hospitalization.

### Limitations

Our study has several limitations. First, follow-up data were not available for all eligible children, creating a potential for bias. However, loss to follow-up was modest (19.8%), and patient characteristics were similar between families completing vs not completing follow-up.^10^ Second, because follow-up surveys occurred 6 months after PICU discharge, recall bias is possible. However, questions prompted respondents to recall both whether any days were missed and the overall number—thus, data were captured even if caregivers could not recall the exact duration of absence. Third, we assumed the school year to occur from September through June. However, this assumption may underestimate the number of school days missed, especially in children older than 5 years. Fourth, surveys did not differentiate school absence during vs after hospitalization, but we were able to estimate postdischarge absence based on length of hospitalization and total school absence. Fifth, data on prehospitalization school and work absence were not available. However, the rate and duration of school absence in children without preexisting comorbidity were only slightly less than the overall cohort rate and duration, suggesting much of the absence observed was attributable to the hospitalization for respiratory failure.^10^

## Conclusions

In this cohort study, nearly 70% of children hospitalized with acute respiratory failure missed school after discharge, for a median duration of nearly 2 school weeks. Similarly, half of primary caregivers missed work after their child’s hospital discharge. The magnitude of school absenteeism suggests a risk for negative downstream educational, financial, and health outcomes.

## Notes

Supplement 1. **eMethods 1.** Follow-up Survey Questions **eMethods 2.** Measurements **eTable 1.** Multivariable Models of Risk Factors to Predict Rate and Duration of School Absence After Hospital Discharge **eTable 2.** School and Work Absence According to RESTORE Treatment Group **eTable 3.** Patient, Family, and Hospitalization Characteristics Across Duration of School Absence Category During and After Hospitalization (n = 454) **eTable 4.** Parent, Family, and Hospitalization Characteristics Across Quartiles of Duration of Postdischarge Work Absence Category in Primary Caregivers (n = 506) **eTable 5.** Patient, Family, and Hospitalization Characteristics Across Quartiles of Duration of Postdischarge Work Absence Category Among Secondary Caregivers (n = 614) **eTable 6.** Postdischarge School Absenteeism by Age Group **eTable 7.** Postdischarge Employment and Work Absenteeism by Age Group

Supplement 2.
Nonauthor Collaborators of the RESTORE Study
