# Supplementary material for: School and Work Absences After Critical Care Hospitalization for Pediatric Acute Respiratory Failure: A Secondary Analysis of a Cluster Randomized Trial
Source: JAMA Netw Open. 2021 Dec 23;4(12):e2140732. doi: 10.1001/jamanetworkopen.2021.40732 (PMC8703244; doi:10.1001/jamanetworkopen.2021.40732)
Supplement: Supplement 1. — eMethods 1. Follow-up Survey Questions eMethods 2. Measurements eTable 1. Multivariable Models of Risk Factors to Predict Rate and Duration of School Absence After Hospital Discharge eTable 2. School and Work Absence According to RESTORE Treatment Group eTable 3. Patient, Family, and Hospitalization Characteristics Across Duration of School Absence Category During and After Hospitalization (n = 454) eTable 4. Parent, Family, and Hospitalization Characteristics Across Quartiles of Duration of Postdischarge Work Absence Category in Primary Caregivers (n = 506) eTable 5. Patient, Family, and Hospitalization Characteristics Across Quartiles of Duration of Postdischarge Work Absence Category Among Secondary Caregivers (n = 614) eTable 6. Postdischarge School Absenteeism by Age Group eTable 7. Postdischarge Employment and Work Absenteeism by Age Group [file jamanetwopen-e2140732-s001.pdf]

Supplementary Online Content

Carlton EF, Donnelly JP, Prescott HC, et al; RESTORE Study Investigators. School and work absences after critical care hospitalization for pediatric acute respiratory failure: a secondary analysis of a cluster randomized trial. *JAMA Netw Open*. 2021;4(12):e2140732. doi:10.1001/jamanetworkopen.2021.40732

eMethods 1. Follow-up Survey Questions

eMethods 2. Measurements

eTable 1. Multivariable Models of Risk Factors to Predict Rate and Duration of School Absence After Hospital Discharge

eTable 2. School and Work Absence According to RESTORE Treatment Group

eTable 3. Patient, Family, and Hospitalization Characteristics Across Duration of School Absence Category During and After Hospitalization (n = 454)

eTable 4. Parent, Family, and Hospitalization Characteristics Across Quartiles of Duration of Postdischarge Work Absence Category in Primary Caregivers (n = 506)

eTable 5. Patient, Family, and Hospitalization Characteristics Across Quartiles of Duration of Postdischarge Work Absence Category Among Secondary Caregivers (n = 614)

eTable 6. Postdischarge School Absenteeism by Age Group

eTable 7. Post-Discharge Employment and Work Absenteeism by Age Group

This supplementary material has been provided by the authors to give readers additional information about their work.

eMethods 1. Follow-Up Survey Questions

<u>Child</u>

Has your child missed any days of daycare, pre-school, or school since being ADMITTED to the hospital roughly 6 months ago? (Include Hospital Stay)

Yes
No
Don't Know
Refused

How many days of daycare, pre-school or school has your child missed (including hospital admission)?

____

<u>Primary and Secondary Caregivers</u>:

While your child was in the hospital, how many days did you have to miss work to be with him/her? [starting with the day of hospital admission until child ultimately discharged home, and including time at a transfer hospital or other medical facility, if applicable]

Not Applicable –Parent does not work
Number___
Don't Know
Refused

While your child was in the hospital, how many days did another person who had been planning to work have to miss work to be with him/her? [starting with the day of hospital admission until child ultimately discharged home, and including time at a transfer hospital or other medical facility, if applicable]

Not Applicable –Parent does not work
Number___
Don't Know
Refused

Since your child was discharged from the hospital, how many days have you had to stay home from work to be with him/her when you been planning to work?

Not Applicable –Parent does not work
Number___
Don't Know
Refused

Since your child was discharged from the hospital, how many days has another person who had been planning to work had to stay home from work to be with him/her?

Not Applicable –Parent does not work
Number___
Don't Know
Refused

<u>Siblings</u>

Does (child's name) have any Siblings?

Yes
No

©2021 Carlton EF et al. *JAMA Network Open*.

Refused

Have (child’s name) siblings missed any days of daycare, pre-school or school due to his/her brother/sister being ill and hospitalized) roughly 6 months ago? (Including days during patient's hospitalization and any days thereafter)

Yes
No
Not Sure
N/A siblings not enrolled in school/not school age
Refused

How many days of daycare, pre-school or school has his/her brothers/sisters had to miss due to [child’s name] illness and hospitalization?

Age	Days During Hospitalization	Days After Hospitalization
____	______	______

©2021 Carlton EF et al. *JAMA Network Open*.

eMethods 2. Measurements

We sought to measure the rate and duration of school absence after hospital discharge. As summer hospitalizations are less likely to result in school absenteeism, we measured the school absence rate only among patients who were discharged during the school year (September-June). We estimated the number of school days missed following discharge by subtracting the length of hospitalization from the total number of school days reported as missed from hospital admission through 6 months post-discharge. We accounted for the 5-day school week (compared to the 7-day calendar week) by multiplying the hospital length of stay by 5/7 (or 0.71). Thus, a patient was considered to have missed school following discharge if the number of school days missed exceeded the total hospital length of stay.

Prior comorbidity status was determined as part of the parent RESTORE study and included prematurity, asthma, seizure disorder, neurologic/neuromuscular disorder, cancer, or known chromosomal abnormality. Prematurity was defined as <36 weeks postmenstrual age. Asthma identified by prescription of bronchodilators or steroids. Cancer included current or prior malignancy diagnosis. Seizure disorders were identified by prescription anticonvulsant medications. Neuromuscular/neurologic disorders included those which put a patient at risk for aspiration.

**eTable 1. Multivariable Models of Risk Factors to Predict Rate and Duration of School Absence After Hospital Discharge**		
**Rate of School Absence (n = 392)**	**Odds Ratio (95% CI)[a]**	***P* Value**
Age group		0.001
0-4 years (reference category)	1.0	
5-8 years	3.2 (1.7-6.2)	
9-12 years	2.6 (1.2-5.4)	
13-18 years	2.4 (1.2-4.8)	
Minority race/ethnicity	0.8 (0.5-1.5)	0.53
Functional impairment (POPC >1) at baseline[b]	1.5 (0.8-2.8)	0.16
Pre-existing comorbidity	1.9 (1.1-3.3)	0.03
Primary diagnosis category		0.39
Pneumonia (reference category)	1.0	
Bronchiolitis or asthma	0.8 (0.5-1.5)	
Acute respiratory failure related to sepsis	1.3 (0.6-2.7)	
Other	1.9 (0.8-4.6)	
Primary caregiver main daily activities		0.001
Working full-time (reference category)	1.0	
Keeping house/raising children	0.3 (0.2-0.6)	
Working part-time	0.8 (0.4-1.6)	
Other (student, retired, disabled)	0.4 (0.2-1.0)	
Number of Siblings	1.0 (0.8-1.2)	0.77
Secondary Caregiver	0.7 (0.4-1.3)	0.26

Abbreviations: CI, confidence interval; POPC, Pediatric Overall Performance Category.
The largest group within each variable was chosen as the reference category.
[a] Odds ratio >1 indicates higher odds of missed school post-discharge. Odds ratios were calculated using logistic regression.
[b] Disability was determined using the Pediatric Overall Performance Category. A score of 1 indicates a good functional status, while a score of 2 or greater represents at least mild disability.

©2021 Carlton EF et al. *JAMA Network Open*.

**eTable 2. School and Work Absence According to RESTORE Treatment Group**			
No./total (%)	**Sedation Protocol**	**Usual Care**	***P* Value**
**Child**			
Any missed days after hospitalization,	136/194 (70.1)	143/205 (69.7)	0.94
No. of missed days, median (IQR)	8.9 (0-26.4)	9.3 (0-27.9)	0.87
**Primary Caregiver**			
Any missed days during hospitalization	225/269 (83.6)	201/237 (84.8)	0.72
No. of missed days during hospitalization, median (IQR)	10 (5-20)	10 (4-20)	0.35
Any missed days after hospitalization	142/269 (52.8)	135/237 (57.0)	0.35
No. of missed days after hospitalization, median (IQR)	2(0-10)	2(0-10)	0.61
**Secondary Caregiver**			
Any missed days during hospitalization	269/344 (78.2)	215/270 (79.6)	0.67
No. of missed days during hospitalization, median (IQR)	5 (2-10)	6 (3-10)	0.76
Any missed days after hospitalization	107/344 (31.1)	86/270 (31.9)	0.84
No. of missed days after hospitalization, median (IQR)	0 (0-2)	0 (0-2)	0.81
Abbreviations: IQR, interquartile range. Missed days among children and caregivers are missed school or work days, respectively.			

©2021 Carlton EF et al. *JAMA Network Open*.

eTable 3. Patient, Family, and Hospitalization Characteristics Across Duration of School Absence Category During and After Hospitalization (n = 454)

No./total (%)	**No Absence (n = 82)**	**Short Absence (n = 129)**	**Medium Absence (n = 125)**	**Long Absence (n = 118)**	***P* Value[a]**
Duration of school absence, median (IQR), d	0 (0-0)	10 (5-12)	25 (20-30)	88.5 (60-112)	<0.001
Range	0-0	1-15	16-40	41-300	
**Patient Characteristics**					
Age at PICU admission, median (IQR), y	2.5 (0.26-10.5)	7.1 (3.6-11.1)	5.8 (3.4-12.1)	9.1 (5.9-12.5)	<0.001
Female	35 (42.7)	58 (45.0)	57 (45.6)	57 (48.3)	0.43
Non-Hispanic white,	43/82 (52.4)	75/129 (58.1)	77/124 (62.1)	78/117 (66.7)	0.03
Functional impairment (POPC >1) at baseline[b]	19 (23.2)	32 (24.8)	39 (31.2)	55 (46.6)	<0.001
Pre-existing comorbidity					
Asthma	8 (9.8)	40 (31.0)	30 (24.0)	13 (11.0)	0.38
Cancer	2 (2.4)	4 (3.1)	5 (4.0)	22 (18.6)	<0.001
Chromosomal abnormality	2 (2.4)	2 (1.5)	8 (6.4)	11 (9.3)	0.005
Prematurity	8 (9.8)	11 (8.5)	11.(8.8)	11 (9.3)	0.98
Neurologic/neuromuscular disorder	6 (7.3)	11 (8.5)	11 (8.8)	22 (18.6)	0.01
Seizure disorder	5 (6.1)	14 (10.9)	18 (14.4)	21 (17.8)	0.01
No known comorbidity	54 (65.9)	62 (48.1)	62 (49.6)	52 (44.1)	0.009
**Family Characteristics**					
Primary caregiver education					
Some high school	2 (3.2)	9 (10.0)	11 (12.1)	8 (10.0)	
High school graduate	14 (22.2)	20 (22.2)	23 (25.3)	15 (18.8)	
Some college or technical school	26 (41.3)	23 (25.6)	21 (23.1)	27 (33.8)	0.76
College graduate/postgraduate	21 (33.3)	38 (42.2)	36 (39.6)	30 (37.5)	
Missing, No.	19	39	24	38	
Primary caregiver main daily activities					
Working full-time	33 (40.7)	62 (48.4)	67 (54.0)	54 (46.6)	
Working part-time	13 (16.1)	19 (14.8)	25 (20.2)	17 (14.7)	
Keeping house/raising children	25 (30.9)	35 (27.3)	24 (19.24	34 (29.3)	0.39
Other (student, retired, disabled)	10 (12.4)	12 (9.4)	8 (6.5)	11 (9.5)	
Missing, No.	1	1	1	2	
Secondary caregiver main daily activities					
Working full-time	45 (71.4)	73 (76.0)	76 (79.2)	79 (84.0)	
Working part-time	6 (9.5)	6 (6.3)	7 (7.3)	6 (6.4)	
Keeping house/raising children	3 (4.8)	6 (6.3)	8 (8.3)	3 (3.2)	0.04
Other (student, retired, disabled)	9 (14.3)	11 (11.5)	5 (5.2)	6 (6.4)	
Missing, No.	19	33	29	24	
Median household income of zip code of residence, median (IQR)	54,059 (44,922-73,200)	54,581 (43,147-78,356)	61,250 (46,519-77,143)	60,257 (45,810-80,833)	0.06
Income category of zip code of residence					

©2021 Carlton EF et al. *JAMA Network Open*.

<$40,000	18 (22.0)	23 (17.8)	17 (13.6)	14 (11.9)	0.08
$40,000 - $79,999	48 (58.4)	74 (57.4)	77 (61.6)	74 (62.7)	
≥$80,000	16 (19.5)	32 (24.8)	31 (24.8)	30 (25.4)	
Duration of missed work during hospitalization of primary caregiver, median (IQR), d	7 (5-10)	7 (5-10)	15 (9-20)	23 (13-35)	<0.001
Duration of missed work after hospitalization of primary caregiver, median (IQR), d	5 (3-7)	5 (2-10)	10 (4-15)	20 (7-40)	<0.001
**Hospitalization Characteristics**					
PRISM III-12 score, median (IQR)[c]	7.5 (3-11)	8 (5-13)	7 (3-14)	10 (5-15)	0.04
Primary diagnosis category					
Bronchiolitis or asthma	29 (35.4)	36 (27.9)	33 (26.4)	8 (6.8)	<0.001
Pneumonia or aspiration pneumonia	34 (41.5)	68 (52.7)	59 (47.2)	66 (55.9)	
Acute respiratory failure related to sepsis	10 (12.2)	15 (11.6)	19 (15.2)	26 (22.0)	
Other[d]	9 (11.0)	10 (7.7)	14 (11.2)	18 (15.5)	
Randomized to *RESTORE* sedation protocol	47 (57.3)	60 (46.5)	57 (45.6)	56 (47.5)	0.24
PICU length of stay, median (IQR), d	8.1 (5.3-14.2)	7.0 (4.6-10.1)	10.9 (7.2-16.4)	15.2 (7.9-25.1)	<0.001
Functional impairment (POPC >1) at discharge[b]	26 (31.7)	42 (32.6)	58 (46.4)	69 (58.5)	<0.001
**Post-hospitalization Outcomes**					
Emergency department use in the 6 months following discharge	26 (31.7)	42 (32.6)	36 (28.8)	49 (41.5)	0.21
Hospital readmission in the 6 months following discharge	16 (19.5)	23 (17.8)	34 (27.2)	54 (45.8)	<0.001

Abbreviations: IQR, interquartile range; PICU, pediatric intensive care unit; POPC, Pediatric Overall Performance Category; PRISM III-12, Pediatric Risk of Mortality III score from first 12 hours in the PICU.

[a] *P* values for comparison across groups were calculated using the chi-square test for trend or the non-parametric test for trend (Jonckheere-Terpstra test) for categorical and continuous variables, respectively. Tertiles for school days missed (short, medium, long absence) were created using whole number of days missed and, as such, it is possible to have unequal numbers of patients in each group.

[b] Disability was determined using the Pediatric Overall Performance Category. A score of 1 indicates a good functional status, while a score of 2 or greater represents at least mild disability.

[c] PRISM III-12 score utilizes physiologic and laboratory variables from the first 12 hours of PICU admission to calculate the overall risk of mortality. A higher score represents a higher risk of mortality.

[d] Other primary diagnoses include pulmonary edema or hemorrhage, pulmonary hypertension, laryngotracheobronchitis, thoracic trauma, pneumothorax, acute chest syndrome, pertussis, exacerbation of lung disease (cystic fibrosis or bronchopulmonary dysplasia), acute respiratory failure related to blood transfusions, acute respiratory failure after bone marrow transplantation, and other.

©2021 Carlton EF et al. *JAMA Network Open*.

**eTable 4. Parent, Family, and Hospitalization Characteristics Across Quartiles of Duration of Postdischarge Work Absence Category in Primary Caregivers (n = 506)**					
No./total (%)	**No Absence (n = 229)**	**Short Absence (n = 123)**	**Medium Absence (n = 70)**	**Long Absence (n = 84)**	***P* Value[a]**
Duration of school absence, median (IQR), d	0 (0-0)	3 (2-5)	10 (8-10)	40 (20-83)	<0.001
Range	0-0	1-5	6-15	16-283	
**Patient Characteristics**					
Age at PICU admission, median (IQR), y	2.0 (0.3-7.7)	3.1 (0.8-7.9)	4.2 (1.0-11.0)	4.9 (0.7-9.5)	0.002
Female	112 (48.9)	57 (46.3)	27 (38.6)	36 (42.9)	0.17
Non-Hispanic white,	127 (55.5)	77 (62.6)	41 (59.4)	56 (68.3)	0.06
Functional impairment (POPC >1) at baseline[b]	35 (15.3)	34 (27.6)	13 (18.6)	32 (38.1)	<0.001
Pre-existing comorbidity					
Asthma	26 (11.4)	24 (19.5)	13 (18.6)	8 (9.5)	0.84
Cancer	11 (4.8)	4 (3.2)	4 (5.7)	13 (15.5)	0.003
Chromosomal abnormality	3 (1.3)	4 (3.3)	3 (4.3)	7 (8.3)	0.003
Prematurity	19 (8.3)	21 (17.1)	10 (14.3)	9 (10.7)	0.33
Neurologic/neuromuscular disorder	13 (5.7)	12 (9.8)	4 (5.7)	11 (13.1)	0.07
Seizure disorder	19 (8.3)	9 (7.3)	4 (5.7)	8 (9.5)	0.97
No known comorbidity	156 (68.1)	68 (55.3)	42 (60.0)	41 (48.8)	0.002
**Family Characteristics**					
Primary caregiver education					
Some high school	14 (8.2)	5 (5.5)	5 (9.6)	3 (5.3)	0.52
High school graduate	38 (22.2)	17 (18.7)	9 (17.3)	15 (26.3)	
Some college or technical school	59 (34.5)	33 (36.3)	13 (25.0)	18 (31.6)	
College graduate/postgraduate	60 (35.1)	36 (39.6)	25 (48.1)	21 (36.8)	
Missing, No.	58	32	18	27	
Secondary caregiver main daily activities					
Working full-time	119 (59.5)	80 (74.1)	47 (88.7)	59 (83.1)	<0.001
Working part-time	23 (11.5)	8 (7.4)	3 (5.7)	3 (4.2)	
Keeping house/raising children	37 (18.5)	16 (14.8)	3 (5.7)	5 (7.0)	
Other (student, retired, disabled)	21 (10.5)	4 (3.7)	0 (0.0)	4 (5.6)	
Missing, No.	29	15	17	13	
Median household income of zip code of residence, median (IQR)	57,755 (45,381-75,286)	55,222 (44,348-74,044)	53,195 (44,823-68,678)	61,972 (45,262-78,401)	0.86
Income category of zip code of residence					
<$40,000	30 (13.1)	23 (18.7)	12 (17.1)	11 (13.1)	0.82
$40,000 - $79,999	148 (64.6)	75 (61.0)	44 (62.9)	54 (64.3)	
≥$80,000	51 (22.3)	25 (20.3)	14 (20.0)	19 (22.6)	
**Hospitalization Characteristics**					
PRISM III-12 score, median (IQR)[c]	7 (4-12)	6 (3-13)	9 (5-13)	8 (4.5-14)	0.35
Primary diagnosis category					
Bronchiolitis or asthma	87 (38.0)	45 (36.6)	17 (24.3)	17 (20.2)	0.004

©2021 Carlton EF et al. *JAMA Network Open*.

Pneumonia or aspiration pneumonia	88 (38.4)	65 (52.9)	40 (57.1)	40 (47.6)	
Acute respiratory failure related to sepsis	34 (14.9)	7 (5.7)	7 (10.0)	15 (17.9)	
Other[d]	20 (8.7)	6 (4.9)	6 (8.6)	12 (14.3)	
Randomized to *RESTORE* sedation protocol	127 (55.5)	56 (45.5)	39 (55.7)	47 (56.0)	0.92
PICU length of stay, median (IQR), d	8.3 (5.8-12.4)	9.2 (5.4-13.8)	12.3 (7.1-20.1)	12.3 (7.3-19.1)	<0.001
Functional impairment (POPC >1) at discharge[b]	48 (21.0)	42 (34.2)	18 (25.7)	48 (57.1)	<0.001
**Post-hospitalization Outcomes**					
Emergency department use in the 6 months following discharge	69 (30.1)	48 (39.0)	34 (48.6)	45 (53.6)	<0.001
Hospital readmission in the 6 months following discharge	57 (24.9)	31 (25.2)	28 (40.0)	43 (51.2)	<0.001

Abbreviations: IQR, interquartile range; PICU, pediatric intensive care unit; POPC, Pediatric Overall Performance Category; PRISM III-12, Pediatric Risk of Mortality III score from first 12 hours in the PICU.

[a] *P* values for comparison across groups were calculated using the chi-square test for trend or the non-parametric test for trend (Jonckheere-Terpstra test) for categorical and continuous variables, respectively.

[b] Disability was determined using the Pediatric Overall Performance Category. A score of 1 indicates a good functional status, while a score of 2 or greater represents at least mild disability.

[c] PRISM III-12 score utilizes physiologic and laboratory variables from the first 12 hours of PICU admission to calculate the overall risk of mortality. A higher score represents a higher risk of mortality.

[d] Other primary diagnoses include pulmonary edema or hemorrhage, pulmonary hypertension, laryngotracheobronchitis, thoracic trauma, pneumothorax, acute chest syndrome, pertussis, exacerbation of lung disease (cystic fibrosis or bronchopulmonary dysplasia), acute respiratory failure related to blood transfusions, acute respiratory failure after bone marrow transplantation, and other.

©2021 Carlton EF et al. *JAMA Network Open*.

eTable 5. Patient, Family, and Hospitalization Characteristics Across Quartiles of Duration of Postdischarge Work Absence Category Among Secondary Caregivers (n = 614)					
No./total (%)	**No Absence (n = 421)**	**Short Absence (n = 65)**	**Medium Absence (n = 70)**	**Long Absence (n = 58)**	***P* Value[a]**
Duration of school absence, median (IQR), d	0 (0-0)	2 (1-2)	4.5 (4-5)	10 (10-40)	<0.001
Range	0-0	1-2	3-5	6-208	
**Patient Characteristics**					
Age at PICU admission, median (IQR), y	1.8 (0.3-7.0)	1.4 (0.2-9.1)	2.0 (0.5-8.2)	3.2 (0.8-11.1)	0.15
Female	201 (47.7)	28 (43.1)	33 (47.1)	34 (58.6)	0.29
Non-Hispanic white,	264 (62.7)	42 (64.6)	41 (58.6)	35 (60.3)	0.54
Functional impairment (POPC >1) at baseline[b]	109 (25.9)	18 (27.7)	27 (38.6)	28 (48.3)	<0.001
Pre-existing comorbidity					
Asthma	56 (13.3)	9 (13.9)	6 (8.6)	4 (6.9)	0.11
Cancer	20 (4.8)	5 (7.7)	2 (2.9)	8 (13.8)	0.051
Chromosomal abnormality	18 (4.3)	6 (9.2)	5 (7.1)	5 (8.6)	0.08
Prematurity	57 (13.5)	8 (12.3)	8 (11.4)	8 (13.8)	0.82
Neurologic/neuromuscular disorder	37 (8.8)	5 (7.7)	7 (10.0)	5 (8.6)	0.93
Seizure disorder	40 (9.5)	4 (6.2)	7 (10.0)	10 (17.2)	0.17
No known comorbidity	247 (58.7)	36 (55.4)	43 (61.4)	30 (51.7)	0.53
**Family Characteristics**					
Primary caregiver education					
Some high school	27 (8.9)	3 (6.8)	2 (4.1)	4 (9.8)	0.81
High school graduate	65 (21.3)	9 (20.5)	6 (12.2)	9 (22.0)	
Some college or technical school	92 (30.2)	13 (29.6)	24 (49.0)	12 (29.3)	
College graduate/postgraduate	121 (39.7)	19 (43.2)	17 (34.7)	16 (39.0)	
Missing, No.	116	21	21	17	
Primary caregiver main daily activities					
Working full-time	156 (37.1)	24 (36.9)	25 (35.7)	31 (53.5)	0.17
Working part-time	172 (40.9)	29 (44.6)	26 (37.1)	17 (29.3)	
Keeping house/raising children	72 (17.1)	11 (16.9)	15 (21.4)	8 (13.8)	
Other (student, retired, disabled)	21 (5.0)	1 (1.5)	4 (5.7)	2 (3.5)	
Median household income of zip code of residence, median (IQR)	58,766 (45,381-76,011)	58,218 (44,803-84,327)	65,906 (49,016-85,170)	67,670 (49,509-86,699)	0.05
Income category of zip code of residence					
<$40,000	64 (15.2)	11 (16.9)	12 (17.1)	8 (13.8)	0.09
$40,000 - $79,999	264 (62.7)	34 (52.3)	36 (51.4)	31 (53.5)	
≥$80,000	93 (22.1)	20 (30.8)	22 (31.4)	19 (32.8)	
**Hospitalization Characteristics**					
PRISM III-12 score, median (IQR)[c]	7 (3-12)	8 (3-12)	6.5 (3-11)	7 (3-15)	0.99
Primary diagnosis category					
Bronchiolitis or asthma	152 (36.1)	25 (38.5)	17 (24.3)	14 (24.1)	0.03
Pneumonia or aspiration pneumonia	182 (43.2)	26 (40.0)	38 (54.3)	27 (46.6)	

©2021 Carlton EF et al. *JAMA Network Open*.

Acute respiratory failure related to sepsis	49 (11.6)	7 (10.8)	9 (12.9)	10 (17.2)	
Other[d]	38 (9.0)	7 (10.8)	6 (8.6)	7 (12.1)	
Randomized to *RESTORE* sedation protocol	237 (56.3)	37 (56.9)	33 (47.1)	37 (63.8)	0.89
PICU length of stay, median (IQR), d	9.0 (6.0-14.5)	10.1 (5.3-19.4)	8.5 (6.3-19.3)	14.4 (8.6-25.1)	0.002
Functional impairment (POPC >1) at discharge[b]	132 (31.4)	21 (32.3)	31 (44.3)	29 (50.0)	<0.001
**Post-hospitalization Outcomes**					
Emergency department use in the 6 months following discharge	136 (32.3)	23 (35.4)	37 (52.9)	33 (56.9)	<0.001
Hospital readmission in the 6 months following discharge	110 (26.1)	22 (33.9)	28 (40.0)	36 (62.1)	<0.001

Abbreviations: IQR, interquartile range; PICU, pediatric intensive care unit; POPC, Pediatric Overall Performance Category; PRISM III-12, Pediatric Risk of Mortality III score from first 12 hours in the PICU.

[a] *P* values for comparisons across groups were calculated using the chi-square test for trend or the non-parametric test for trend (Jonckheere-Terpstra test) for categorical and continuous variables, respectively.

[b] Disability was determined using the Pediatric Overall Performance Category. A score of 1 indicates a good functional status, while a score of 2 or greater represents at least mild disability.

[c] PRISM III-12 score utilizes physiologic and laboratory variables from the first 12 hours of PICU admission to calculate the overall risk of mortality. A higher score represents a higher risk of mortality.

[d] Other primary diagnoses include pulmonary edema or hemorrhage, pulmonary hypertension, laryngotracheobronchitis, thoracic trauma, pneumothorax, acute chest syndrome, pertussis, exacerbation of lung disease (cystic fibrosis or bronchopulmonary dysplasia), acute respiratory failure related to blood transfusions, acute respiratory failure after bone marrow transplantation, and other.

©2021 Carlton EF et al. *JAMA Network Open*.

**eTable 6. Postdischarge School Absenteeism by Age Group**					
No./total (%)	Age 0-4 Years<br>n=152	Age 5-9 Years<br>n=112	Age 10-12 Years<br>n=58	Age 13-18 Years<br>n=77	P Value[a]
Any missed days after hospitalization	83 (54.6%)	92 (82.1%)	45 (77.6%)	59 (76.2%)	<0.001
No. of missed days, median (IQR)[a]	3.6 (0-14.6)	11.4 (2.3-42.0)	15.1 (1.3-57.9)	14.3 (1.6-36.4)	<0.001
Chronic absenteeism after hospitalization	38 (25.0%)	49 (43.8%)	29 (50.0%)	37 (48.1%)	<0.001

[a] *P* values for comparison across groups were calculated using the chi-square test for trend or the non-parametric test for trend (Jonckheere-Terpstra test) for categorical and continuous variables, respectively

©2021 Carlton EF et al. *JAMA Network Open*.

`

eTable 7. Postdischarge Primary Caregiver Employment and Work Absenteeism by Age Group					
No./total (%)	Primary Caregivers of Children Age 0-4 Years n=628	Primary Caregivers of Children Age 5-9 Years n=143	Primary Caregivers of Children Age 10-12 Years n=79	A Primary Caregivers of Children Age 13-18 Years n=110	P Value[a]
Primary caregiver employed	308 (63.4%)	82(62.9%)	49 (67.1%)	67 (66.4%)	0.003
Any missed days after hospitalization[b]	156 (50.7%)	52 (63.4%)	35 (71.4%)	59 (50.8%)	0.20

[a] *P* values for comparison across groups were calculated using the chi-square test for trend or the non-parametric test for trend (Jonckheere-Terpstra test) for categorical and continuous variables, respectively. Comparison for age groups 0-4, 5-9, 10-12, 13-18 years.

[b] Total based on number of primary caregivers who reported employment

Additionally, there were 357 primary caregivers of children less the 1 year of age. Of these, 164 (45.9%) were employed outside of the home, among whom 76 missed work following their child's discharge.

©2021 Carlton EF et al. *JAMA Network Open*.
