# Supplementary material for: School and Work Absences After Critical Care Hospitalization for Pediatric Acute Respiratory Failure: A Secondary Analysis of a Cluster Randomized Trial
Source: JAMA Netw Open. 2021 Dec 23;4(12):e2140732. doi: 10.1001/jamanetworkopen.2021.40732 (PMC8703244; doi:10.1001/jamanetworkopen.2021.40732)
Supplement: Supplement 2. — Nonauthor Collaborators of the RESTORE Study [file jamanetwopen-e2140732-s002.pdf]

Supplemental Online Content: Nonauthor Collaborators

*Indicates required information. Only first name, last name, and suffix will appear in PubMed.

*Group Name(s): The RESTORE study investigators							
***First Name and Middle Initial(s)**	***Last Name**	***Suffix (eg, Jr, III)**	Academic Degrees	Institution	Location (city, state/province, country)	Role or Contribution, eg, chair, principal investigator	Group (if more than 1 Group listed in the byline) and/or Subgroup (eg, Steering Committee)
Martha A.Q.	Curley			School of Nursing and the Perelman School of Medicine, University of Pennsylvania	Philadelphia, PA	Principal Investigator	
David	Wypij			Department of Biostatistics, Harvard T.H. Chan School of Public Health; Department of Pediatrics, Harvard Medical School; Department of Cardiology, Boston Children's Hospital	Boston, MA	Principal Investigator- Data Coordinating Center	
Geoffrey L.	Allen			Children's Mercy Hopsital	Kansas City, MO		
Derek C.	Angus			Clinical Research, Investigation and Systems Modeling of Acute Illness Center	Pittsburgh, PA		
Lisa A.	Asaro			Department of Cardiology, Boston Children's Hospital	Boston, MA		
Judy A.	Ascenzi			The Johns Hopkins Hospital	Baltimore, MD		
Scot T.	Bateman			University of Massachusetts Memorial Children's Medical Center	Worcester, MA		
Santiago	Borasino			Children's Hospital of Alabama	Birmingham, AL		
Cindy	Darnell Bowens			Children's Medical Center of Dallas	Dallas, TX		
G. Kris	Bysani			Medical City Children's Hospital	Dallas, TX		
Ira M.	Cheifetz			Duke Children's Hospital	Durham, NC		
Allison S.	Cowl			Connecticut Children's Medical Center	Hartford, CT		
Brenda L.	Dodson			Department of Pharmacy, Boston Children's Hopsital	Boston, MA		
E. Vincent S.	Faustino			Yale-New Haven Chidlren's Hospital	New Haven, CT		

*Indicates required information. Only first name, last name, and suffix will appear in PubMed.

*First Name and Middle Initial(s)	*Last Name	*Suffix (eg, Jr, III)	Academic Degrees	Institution	Location (city, state/province, country)	Role or Contribution, eg, chair, principal investigator	Group (if more than 1 Group listed in the byline) and/or Subgroup (eg, Steering Committee)
Lori D.	Fineman			University of California San Francisco Benioff Children's Hospital At San Francisco	San Francisco, CA		
Heidi R.	Flori			University of California San Francisco Benioff Children's Hospital At San Francisco	San Francisco, CA		
Linda S.	Franck			University of California San Francisco School of Nursing	San Francisco, CA		
Rainer G.	Gedeit			Department of Pediatrics, Medical College of Wisconsin	Milwaukee, WI		
Mary Jo C.	Grant			Primary Children's Hospital	Salt Lake Cit, UT		
Andrea L.	Harabin			National Heart, Lung, and Blood Institute, National Institutes of Health	Bethesda, MD		
Catherine	Haskins-Kiefer			Florida Hospital for Children	Orlando, FL		
James H.	Hertzog			Neumours/Alfred I. duPont Hospital for Children	Wilmington, DE		
Larissa	Hutchins			The Children's Hospital of Philadelphia	Philadelphia, PA		
Aileen L.	Kirby			Oregon Health and Science University Doernbecher Children's Hospital	Portland, OR		
Ruth M.	Lebet			School of Nursing, University of Pennsylvania	Philadelphia, PA		
Michael A.	Matthay			University of California at San Francisco School of Medicine	San Francisco, CA		
Gwenn E.	McLaughlin			Holtz Children's Hospital, Jackson Health System	Miami, FL		
JoAnne E.	Natale			University of California Davis Children's Hospital	Sacramento, CA		
Phineas P.	Oren			St. Louis Children's Hospital	St. Louis, MO		

Supplemental Online Content: Nonauthor Collaborators

*Indicates required information. Only first name, last name, and suffix will appear in PubMed.

***First Name and Middle Initial(s)**	***Last Name**	***Suffix (eg, Jr, III)**	Academic Degrees	Institution	Location (city, state/province, country)	Role or Contribution, eg, chair, principal investigator	Group (if more than 1 Group listed in the byline) and/or Subgroup (eg, Steering Committee)
Nagendra	Polavarapu			Advocate Chidlren's Hospital-Oak Lawn	Oak Lawn, IL		
James B.	Schneider			Cohen Children's Medicine Center of New York	Hyde Park, NY		
Adam J.	Schwarz			Children's Hospital of Orange County	Orange, CA		
Thomas P.	Shanley			C.S. Mott Children's Hospital at the University of Michigan	Ann Arbor, MI		
Shari	Simone			University of Maryland Medical Center	Baltimore, MD		
Lewis P.	Singer			The Children's Hospital at Montefiore	Bronx, NY		
Lauren R.	Sorce			Ann & Robert H. Lurie Children's Hospital of Chicago	Chicago, IL		
Edward J.	Truemper			Children's Hospital and Medical Center	Omaha, NE		
Michele A.	Vander Heyden			Children's Hospital at Dartmouth	Dartmouth, NH		
R. Scott	Watson			Center for Child, Health, Behavior and Development, Seattle Children's Research Institute	Seattle, WA		
Claire R.	Wells			University of Arizona Medical Center	Tucson, AZ		
